# CHARACTERIZATION OF PATIENTS TREATED AT A UNIVERSITY OUTPATIENT CLINIC WITH FUNCTIONAL DYSPEPSIA AND ITS CORRELATION WITH MENTAL DISORDERS

**DOI:** 10.1590/S0004-2803.24612026-025

**Published:** 2026-10-26

**Authors:** Daniella Ribeiro Einstoss KORMAN, Jordana de Castro HONORATO, Júlia Brunetti Teles de MELO, Lucas Leonardo Knupp dos SANTOS

**Affiliations:** * Faculdade Ciências Médicas de Minas Gerais, Belo Horizonte, Minas Gerais, Brasil.

**Keywords:** Functional dyspepsia, social determinants of health, mental disorders, Dispepsia funcional, determinantes sociais de saúde, transtornos mentais

## Abstract

**Background:**

Functional dyspepsia is a common gastrointestinal disorder in clinical practice, characterized by chronic dyspeptic symptoms in the absence of identifiable organic disease, and is currently classified as a disorder of gut-brain interaction. Evidence suggests a significant association between functional dyspepsia and mental disorders, particularly anxiety and depression; however, this relationship remains insufficiently explored in national studies.

**Objective:**

To characterize the sociodemographic and clinical profile of patients treated at a university outpatient gastrointestinal care clinic with functional dyspepsia and to analyze its correlation with mental disorders.

**Methods:**

This was a cross-sectional observational study based on the analysis of medical records of patients diagnosed with functional dyspepsia according to the Rome IV criteria, treated at a university outpatient clinic in the southeast of Brazil. Sociodemographic data, lifestyle habits, clinical comorbidities, presence of concomitant mental disorders, and information on clinical evolution were collected. Statistical analyses were performed to evaluate associations between functional dyspepsia, mental disorders and clinical variables.

**Results:**

The sample consisted of 135 patients, predominantly women, with a higher prevalence of patients over 60 years of age. Most individuals were sedentary, non-smokers, and non-alcohol users, with systemic arterial hypertension being the most frequent clinical comorbidity. Mental disorders were present in nearly half of the patients, with generalized anxiety disorder and major depressive disorder being the most prevalent diagnoses, either isolated or in combination. No statistically significant associations were observed between mental disorders and most sociodemographic or clinical variables, except for obesity. During outpatient follow-up, a substantial proportion of patients with functional dyspepsia and concomitant mental disorders showed partial or complete improvement of dyspeptic symptoms.

**Conclusion:**

The findings demonstrate a high frequency of mental disorders among patients with functional dyspepsia treated at a university outpatient clinic, reinforcing the multifactorial nature of this condition. The observed association and the clinical improvement identified in a subset of patients undergoing multidisciplinary follow-up highlight the importance of integrating gastroenterology and mental health care in the management of functional dyspepsia, contributing to a more comprehensive and interdisciplinary care of the patient.

## HIGHLIGHTS

• Functional dyspepsia showed a higher prevalence among women and among individuals aged over 60 years old treated at a university outpatient clinic.

• Mental disorders were present in approximately 40% of patients with functional dyspepsia, with generalized anxiety disorder and major depressive disorder being the most frequent diagnoses.

• No significant associations were observed between mental disorders and most sociodemographic or clinical variables, except for obesity.

• A substantial proportion of patients with functional dyspepsia and concomitant mental disorders showed partial or complete improvement of dyspeptic symptoms during multidisciplinary follow-up.

## RESUMO

**Contexto:**

A dispepsia funcional é um distúrbio gastrointestinal frequente na prática clínica, caracterizado pela presença de sintomas dispépticos crônicos sem causa orgânica identificável, sendo atualmente classificada como um distúrbio da interação cérebro-intestino. Evidências sugerem associação significativa entre dispepsia funcional e transtornos mentais, especialmente ansiedade e depressão, embora essa relação ainda seja pouco explorada em estudos nacionais.

**Objetivo:**

Caracterizar o perfil sociodemográfico e clínico de pacientes atendidos na linha de cuidados gastrointestinal de um ambulatório universitário com dispepsia funcional e analisar sua correlação com transtornos mentais.

**Métodos:**

Trata-se de um estudo observacional transversal, realizado a partir da análise de prontuários de pacientes diagnosticados com dispepsia funcional conforme os critérios de Roma IV, atendidos em um ambulatório universitário. Foram coletadas informações sociodemográficas, hábitos de vida, comorbidades clínicas, presença de transtornos mentais concomitantes e dados sobre a evolução clínica dos pacientes. As análises estatísticas foram realizadas para avaliar associações entre dispepsia funcional, transtornos mentais e variáveis clínicas.

**Resultados:**

A amostra consistiu em 135 pacientes, composta predominantemente por mulheres, com maior prevalência de pacientes acima de 60 anos. A maioria dos indivíduos era sedentária, não tabagista e não etilista, sendo a hipertensão arterial sistêmica a comorbidade clínica mais frequente. Transtornos mentais estavam presentes em quase metade dos pacientes, destacando-se o transtorno de ansiedade generalizada e o transtorno depressivo maior, isoladamente e em associação. Não foram observadas associações estatisticamente significativas entre transtornos mentais e a maioria das variáveis sociodemográficas e clínicas, com exceção da obesidade. Durante o acompanhamento ambulatorial, parte expressiva dos pacientes com dispepsia funcional e transtornos mentais concomitantes apresentou melhora total ou parcial dos sintomas dispépticos.

**Conclusão:**

Os achados evidenciam elevada frequência de transtornos mentais em pacientes com dispepsia funcional atendidos em um ambulatório universitário, reforçando o caráter multifatorial dessa condição. A associação observada e a melhora clínica identificada em parte dos pacientes acompanhados de forma multidisciplinar destacam a importância da integração entre gastroenterologia e saúde mental no manejo da dispepsia funcional, contribuindo para um cuidado mais abrangente e centrado no paciente.

## INTRODUCTION

Functional dyspepsia is a gastrointestinal disorder characterized by chronic symptoms centered in the epigastrium, including pain or discomfort, early satiety, and postprandial fullness, in the absence of any organic, systemic, or metabolic disease capable of explaining the patient’s symptoms^1^ ^,^ ^2^. Functional dyspepsia affects approximately 5-11% of the global population and accounts for nearly 80% of patients presenting with dyspeptic symptoms in clinical practice^3^ ^,^ ^4^. Although its etiology remains unclear, its pathophysiology is recognized as multifactorial. The overlap of symptoms and gastroduodenal motor and sensory abnormalities emphasizes the heterogeneity of functional dyspepsia^5^ ^,^ ^6^.

In 2016, functional gastrointestinal disorders were redefined by the Rome IV criteria as disorders of gut-brain interaction. This group currently comprises 33 conditions and affects up to 40% of the world’s population, with functional dyspepsia and irritable bowel syndrome being the most prevalent^7^. Communication between the central nervous system (CNS) and the gastrointestinal tract occurs through the gut-brain axis, a bidirectional network integrating neural, endocrine, and immune signaling pathways. This interaction is facilitated by the shared embryological origin of the CNS and the enteric nervous system, as well as by common receptors and neurotransmitters - such as gamma-aminobutyric acid (GABA), norepinephrine, serotonin and dopamine - that modulate both gastrointestinal function and emotional and behavioral responses. Psychological factors may trigger and perpetuate dyspeptic symptoms by promoting dysregulation of the hypothalamic-pituitary-adrenal axis, increasing corticotropin and cortisol release, and activating the autonomic nervous system, thereby leading to alterations in gastrointestinal motility, permeability, and visceral sensitivity. The gastrointestinal tract and the CNS maintain a complex and bidirectional interaction mediated by the microbiota-gut-brain axis^6^ ^,^ ^8^. Among digestive disorders, functional dyspepsia has been associated with generalized anxiety disorder, somatization, and major depressive disorder^3^ ^,^ ^9^ ^,^ ^10^ ^,^ ^11^. A prospective cohort study conducted in 2022 at a tertiary gastrointestinal referral center in the United States evaluated 128 individuals with functional dyspepsia and demonstrated an association between changes in anxiety disorder severity and variations in dyspeptic symptom severity^12^.

Recent Brazilian literature corroborates the association between functional dyspepsia and mental disorders, particularly anxiety and depression, with a negative impact on quality of life^9^ ^-^ ^11^. Data from the Rome Foundation Global Epidemiology Study, which included a representative Brazilian sample, indicate that Brazilian individuals with disorders of gut-brain interaction (including functional dyspepsia) exhibit a significantly higher prevalence of anxiety and depressive symptoms compared with those without such disorders. Furthermore, these patients report poorer health-related quality of life. In a clinical study involving 348 patients, the presence of depression was associated with an increased risk of functional dyspepsia after adjustment for sociodemographic variables (OR ≈ 3.13)^13^. In 2019, a cross-sectional study including 859 dyspeptic patients referred for upper gastrointestinal endoscopy in Sergipe, Brazil, evaluated epidemiological and clinical aspects related to functional dyspepsia. More than half of the individuals with functional dyspepsia exhibited anxiety symptoms and one-quarter reported depressive symptoms^10^.

Despite these findings, national studies characterizing functional dyspepsia and exploring its risk factors, as well as other psychosocial determinants that may coexist with the disorder, remain limited. These include mood disorders such as bipolar disorder; neurotic disorders, including phobic-anxiety and obsessive-compulsive disorders; and behavioral syndromes such as eating disorders. Brazilian studies providing direct evidence on the effectiveness of specific interventions, such as psychotherapy or antidepressant therapy, are also lacking. Current evidence on these approaches is largely derived from international studies and meta-analyses. Therefore, further research in this field is essential, as such evidence may support the development of more effective multidisciplinary management strategies and contribute to the implementation of screening protocols better suited to the national context.

Accordingly, it became necessary to conduct a Brazilian study aimed at characterizing patients with functional dyspepsia treated within the gastrointestinal care pathway of a secondary healthcare unit and at analyzing the coexistence and influence of different mental disorders on the dyspeptic clinical presentation. The importance of this investigation lies in the substantial prevalence of functional dyspepsia among gastroduodenal complaints and, consequently, in the need for appropriate and individualized treatment strategies. By identifying correlations between this gastrointestinal disorder and specific mental health conditions, multidisciplinary interventions involving gastroenterology, psychiatry, and psychology could be developed. Effective management of a psychiatric disorder may contribute to improvement in dyspeptic symptoms and vice versa, thereby ensuring comprehensive, patient-centered care grounded in a biopsychosocial approach.

## METHODS

This was a cross-sectional analytical observational study conducted during the Institutional Undergraduate Research Scholarship Program (Programa Institucional de Bolsas de Iniciação Científica - PROBIC) of the Faculdade Ciências Médicas de Minas Gerais, from February 2025 to February 2026. The project is part of an umbrella study approved by the Research Ethics Committee (approval number: 5.894.420) and conducted in accordance with the Brazilian General Data Protection Law. The study was based on the review of medical records of adult patients of both sexes treated at the gastrointestinal care pathway of the Ciências Médicas de Minas Gerais Outpatient Clinic, in Belo Horizonte, Minas Gerais, Brazil.

Inclusion criteria were: enrollment in the gastrointestinal care pathway of the Ciências Médicas de Minas Gerais Outpatient Clinic; age 18 years or older; diagnosis of functional dyspepsia, defined as dyspeptic symptoms with exclusion of structural or metabolic etiologies. Exclusion criteria were: diagnosis of another concomitant gastrointestinal disorder; oncological disease; pregnancy or breastfeeding; history of upper gastrointestinal tract surgery; age 80 years or older. The flowchart shown in Figure 1 details the patients’ selection process in a dynamic way, including the number of initially screened records, the exclusions and the final sample composition.

**FIGURE 1:**
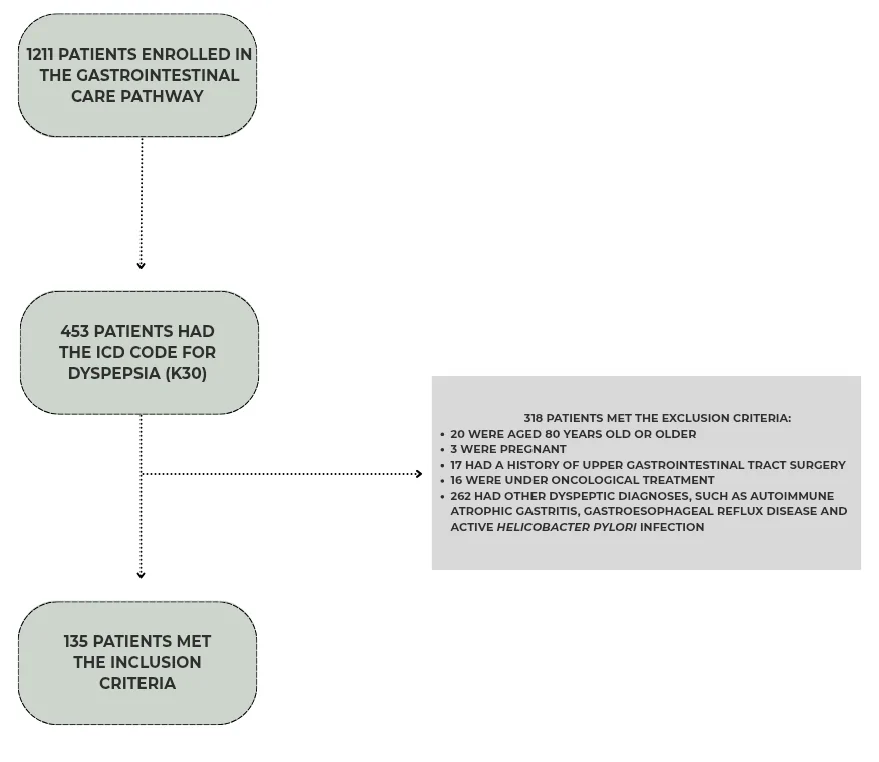
Flowchart presenting the participants’ selection process.

The quantitative variable collected was age. Qualitative variables included sex, city of residence, marital status, alcohol consumption, active smoking, smoking history, physical activity, comorbidities, mental disorders and clinical evolution. The comorbidities evaluated were diabetes mellitus, systemic arterial hypertension, dyslipidemia, obesity and hypothyroidism. Data were collected using the MV PEP system, the institutional electronic medical record system, and a Google Forms questionnaire to transfer the variables of interest from the medical records to an Excel spreadsheet for subsequent statistical analysis.

The primary objective was to characterize the profile of patients with functional dyspepsia treated at the gastrointestinal care pathway of the outpatient clinic using the previously selected variables. In addition, the presence of concomitant mental disorders was assessed by investigating ICD codes for Mental and Behavioral Disorders (F00-F99) recorded in the patients’ medical records. The identification of psychiatric diagnoses was based exclusively on information documented during routine clinical care. No standardized psychiatric scales or structured diagnostic interviews were applied specifically for the purposes of this study. For patients meeting these criteria, the correlation between psychiatric and gastrointestinal disorders was evaluated, as well as changes in dyspeptic symptoms following a multidisciplinary approach involving gastroenterology and psychiatry or psychology.

Regarding statistical methodology, frequency tables and one figure were used to characterize the dataset. Qualitative variables were described using absolute and relative frequencies. Quantitative variables were expressed as means and interquartile ranges. Associations between qualitative variables were assessed using Fisher’s exact test or the chi-square test of independence. For quantitative variables, the Shapiro-Wilk test was applied to assess normality, followed by Student’s *t* test to evaluate relationships between quantitative variables and analysis of variance (ANOVA) to test differences among the means of three or more groups. When variables did not show a normal distribution, nonparametric tests were applied. Data analysis was performed using R software, version 4.5.

## RESULTS

Regarding data collection, medical records of 1,211 patients enrolled in the gastrointestinal care pathway were reviewed according to the ICD code of interest: Dyspepsia (K30), covering the period from June 2015 to September 2025. Of these records, 453 had the ICD code for dyspepsia (K30). Subsequently, records meeting the inclusion criteria were selected, resulting in a total of 135 patients. These were analyzed according to the variables previously selected by the researchers. Thus, 67 individuals (49.6%) were aged over 60 years, while 62 (45.9%) were between 35 and 59 years of age. Regarding sex, 107 participants (79.3%) were women. In addition, 91.9% of the patients were residents of Belo Horizonte, with the remaining participants evenly distributed across 11 other cities in the state of Minas Gerais, predominantly in the central region of the state. Concerning marital status, 51 individuals were married (37.8%), followed by 44 single participants (32.6%). The statistics regarding the socio-demographic variables are presented in Table 1.

**TABLE 1:** Socio-demographic variables.

Socio-demographic variables	Absolute Frequency	Relative Frequency
Age 60-79 years old	67	49.6%
Age 35-59 years old	62	45.9%
Age 18-34 years old	6	4.4%
Female	107	79.3%
Male	28	20.7%
Residence in belo horizonte	124	91.9%
Married	51	37.8%
Single	44	32.6%
Divorced	23	17%
Widowed	16	11.9%
Common-law marriage	1	0.7%
Sedentary	88	65.2%
Physically active	45	33.3%
Non smoker	90	66.7%
Active smoker	29	21.5%
Former smoker	16	11.9%
Smoking history of >20 pack-years	27	60%
Smoking history of <20 pack-years	18	40%
Abstains from alcohol consumption	94	69.6%
Social alcohol consumption	27	20%
Alcohol use disorder	6	4.4%

Regarding physical activity, 88 participants were sedentary. Moreover, 66.7% were neither current smokers nor former smokers. Among those who had a history of smoking, including both former and current smokers, a smoking exposure greater than 20 pack-years predominated (60%). Most of the 135 individuals were non-alcohol users (69.6%), as shown in Table 1 as well. The majority of the sample consisted of non-diabetic (71.9%), and non-dyslipidemic (53.3%) patients, respectively. Furthermore, according to Table 2, the prevalence of non-obese (68.1%) and non-hypothyroid individuals (87.4%) was also higher. However, there were more patients with systemic arterial hypertension (63.7%) than non-hypertensive individuals (36.3%).

**TABLE 2:** Comorbidities.

Comorbidities	Absolute Frequency	Relative Frequency
Systemic arterial hypertension	86	63.7%
Diabetes mellitus	38	28.1%
Dyslipidemia	63	46.7%
Obesity	43	31.9%
Hypothyroidism	17	12.6%
Mental disorder	55	40.7%

Regarding the association with mental disorders, 80 patients with functional dyspepsia (59.3%) had no diagnosis of any mental disorder, whereas 55 participants (40.7%) had a diagnosis of at least one mental disorder. Among these patients, 23 (41.8%) had generalized anxiety disorder (GAD) alone, while 17 (31.0%) had major depressive disorder (MDD) alone. In addition, 10 patients (18.2%) had concomitant GAD and MDD. One patient (1.8%) was diagnosed with MDD and schizophrenia; one (1.8%) had GAD, MDD, and binge eating disorder; one (1.8%) had GAD and an unspecified mood disorder; one (1.8%) had MDD and antisocial personality disorder; and one (1.8%) had GAD and panic disorder. These data are detailed in Table 3.

**TABLE 3:** Mental disorder distribution.

Mental disorder distribution	Absolute Frequency	Relative Frequency
Gad only	23	41.8%
Mdd only	17	31%
Gad + mdd	10	18.2%
Mdd + schizophrenia-	1	1.8%
Gad + mdd + binge eating disorder	1	1.8%
Gad + unspecified mood disorder	1	1.8%
Mdd + antisocial personality disorder	1	1.8%
Gad + panic disorder	1	1.8%
Total	55	100%

Medical records of patients with functional dyspepsia and concomitant mental disorder who were enrolled in the gastrointestinal care pathway of the Ciências Médicas Outpatient Clinic were reviewed, totaling 55 patients. During the data collection period, these individuals were under regular follow-up in gastroenterology as well as psychiatry and/or psychology. Thus, clinical records from both gastroenterologists and psychiatrists and/or psychologists were analyzed over time to assess whether improvement in dyspeptic symptoms occurred in parallel with improvement in psychiatric symptoms. The assessment of clinical improvement was based solely on the report described in medical records, since the research was purely observational, using only the reading and interpretation of medical records and appointments carried out by other professionals: gastroenterologists, psychiatrists or psychologists. Therefore, the researchers did not use any instruments to assess the quality of life of patients with functional disorders or the improvement of dyspeptic and/or psychiatric symptoms. As shown in Table 4, regarding clinical evolution, 15 patients (27.3%) showed complete improvement, 12 (21.8%) showed partial improvement, 13 (23.6%) showed no improvement, and for 15 patients (27.3%) there were insufficient data to determine improvement in dyspeptic symptoms. All patients with mental disorders were using at least one psychiatric medication. Among those who showed complete improvement in dyspeptic symptoms, 40% were using selective serotonin reuptake inhibitors (SSRIs), with sertraline being the most prevalent medication in this class, followed by tricyclic antidepressants, especially amitriptyline. Among those patients who showed partial improvement, SSRIs were also more prevalent (58.3%), with sertraline and escitalopram equivalent in number. As for those who did not achieve improvement of functional dyspepsia, the class of medications that stood out was tricyclic antidepressants, with nortriptyline and amitriptyline tied (46.1%). In second place, dual antidepressants, SSRIs, and mirtazapine had the same prevalence.

**TABLE 4:** Clinical evolution.

Clinical evolution	Absolute Frequency	Relative Frequency
Insufficient data	15	27.3%
Complete improvement	15	27.3%
No improvement	13	23.6%
Partial improvement	12	21.8%
Total	55	100%

 Statistical analysis of the associations between mental disorders and clinical variables did not demonstrate significant relationships with most of the comorbidities evaluated (Table 5), with the exception of obesity (*P*=0.036; Cramér’s V = 0.188).

**TABLE 5:** Associations with the mental disorder variable.

Associations with the mental disorder variable	Test statistic	***P*-Value**	Cramér’s V
Marital status	2.189861	0.7736132	-
Physical activity	1.363636	0.6541729	-
Active smoking	1.827905	0.4222889	-
Alcohol consumption	8.242664	0.05647176	-
Diabetes mellitus	0.09766685	0.8565717	-
Systemic arterial hypertension	0.04805411	0.8655672	-
Dyslipidemia	1.790675	0.2103948	-
Obesity	4.783241	0.03648176	0.188232335694153
Hypothyroidism	0.4037886	0.6021989	-
Clinical evolution	2.889325	0.6216892	-

## DISCUSSION

In this study, a detailed characterization of patients with functional dyspepsia treated within the gastrointestinal care pathway of a university outpatient clinic was performed, in addition to an analysis of the coexistence of mental disorders in this population, an aspect that remains insufficiently explored in Brazilian studies. The findings reinforce the multifactorial nature of the disease and its close relationship with psychosocial factors, in accordance with the gut-brain interaction disorder model established by the Rome IV criteria^7^ ^,^ ^14^.

The predominance of female patients (79.3%) observed in this study is consistent with data reported in the international literature, which indicate a higher prevalence of functional dyspepsia among women^3^ ^,^ ^4^. This difference may be explained by multiple factors, including greater visceral sensitivity, hormonal influences on gastric motility, and increased vulnerability to anxiety and depressive disorders. In addition, women tend to seek health care services more frequently, which may contribute to greater female representation in clinical studies and influence sample composition.

Regarding age distribution, a higher concentration of patients in older age groups was observed, particularly those over 60 years of age (49.6%). Although functional dyspepsia can affect younger adults, studies suggest that the accumulation of comorbidities, chronic medication use, and prolonged exposure to psychosocial stressors throughout life may contribute to the persistence or intensification of dyspeptic symptoms at more advanced ages^1^ ^,^ ^2^. This age profile may also help explain the high prevalence of systemic arterial hypertension observed in the sample (63.7%).

With respect to lifestyle habits, most patients were sedentary (65.2%), non-smokers (66.7%), and non-alcohol users (69.6%). These findings suggest that, in this specific population, traditional behavioral factors may have a limited isolated impact on the manifestation of functional dyspepsia, reinforcing the hypothesis that central and psychosocial mechanisms play a more relevant role in the origin and maintenance of symptoms. This interpretation is consistent with studies demonstrating that the intensity of dyspeptic symptoms does not always correlate directly with traditional risk factors, but rather with emotional and perceptual aspects^5^ ^,^ ^8^.

One of the most relevant findings of this study was the high prevalence of mental disorders among patients with functional dyspepsia, present in 40.7% of the sample. Generalized anxiety disorder and major depressive disorder, identified in 65.5% and 54.5% of affected patients, respectively, were the most frequent diagnoses, occurring either in isolation or in combination. These results corroborate international data showing a higher prevalence of these psychiatric disorders in patients with functional gastrointestinal disorders compared with the general population^3^ ^,^ ^4^. However, these findings should be interpreted in light of the characteristics of the evaluated population. Because this study was conducted in a university outpatient referral center, the sample may include a greater proportion of patients with more severe, persistent or difficult-to-manage symptoms who are more likely to be referred for specialized care. Therefore, the frequency of associated psychiatric disorders observed in this study may reflect the profile of patients treated at this institution and should not be generalized to all individuals with functional dyspepsia.

The association between functional dyspepsia and anxiety and depressive disorders may be explained by alterations in central modulation of visceral pain, gastric hypersensitivity, and dysregulation of the hypothalamic-pituitary-adrenal axis. Neurobiological evidence indicates that individuals with these disorders exhibit increased activation of brain regions involved in emotional and sensory processing of pain, which amplifies the perception of gastrointestinal symptoms and is associated with greater severity of both dyspeptic and anxiety-depressive symptoms^6^ ^,^ ^8^.

In addition, autonomic dysfunction and persistent activation of the hypothalamic-pituitary-adrenal axis, frequently associated with chronic stress, contribute to changes in gastric motility, increased intestinal permeability, and the perpetuation of low-grade inflammation mechanisms that reinforce visceral hypersensitivity^6^ ^,^ ^14^. Thus, dyspeptic symptoms may be exacerbated by mental disorders, while the chronic presence of gastrointestinal symptoms may act as a perpetuating or aggravating factor for psychological distress, characterizing a bidirectional relationship between the gut-brain axis and functional gastrointestinal disorders.

Among the evaluated comorbidities, only obesity showed a significant association with mental disorders. The association between mental disorders and obesity has been described in previous studies and may reflect shared mechanisms, such as chronic low-grade inflammation, neuroendocrine alterations, and dysfunctional eating patterns^12^. Although the observed effect size was modest, this finding remains clinically relevant, as it highlights the potential interaction between metabolic and psychological factors in patients with functional dyspepsia.

Regarding clinical evolution, a significant proportion of patients with functional dyspepsia and concomitant mental disorders showed partial or complete improvement of dyspeptic symptoms during multidisciplinary follow-up. This result is consistent with prospective studies demonstrating that improvement in anxiety or depressive symptoms is associated with reduced severity of functional dyspepsia^12^. Another aspect that deserves consideration is the potential influence of psychotropic medications during follow-up. Antidepressants and anxiolytics may affect gastrointestinal motility and visceral symptom perception and could therefore have contributed, at least partially, to the clinical changes observed over time, especially the serotonin reuptake inhibitors (SSRIs) class of antidepressants. This finding reinforces the importance of an integrated approach involving gastroenterology, psychiatry, and psychology, particularly in patients with gastrointestinal symptoms refractory to pharmacological treatment.

When the analysis is expanded to follow-up data from international reference centers, functional dyspepsia appears to follow a chronic and fluctuating course, with a tendency toward symptom persistence over time. Long-term cohort studies indicate that approximately 40% to 50% of patients remain symptomatic after five years, although periods of partial improvement or transient remission may occur, without progression to structural organic disease^15^ ^,^ ^16^. Evidence from specialized centers also suggests that the presence of mental disorders, particularly anxiety and depression, is associated with poorer clinical outcomes, higher symptom recurrence, and increased healthcare utilization. In contrast, integrated therapeutic approaches have been associated with more favorable outcomes^15^.

It is noteworthy that the scarcity of national longitudinal follow-up data limits the assessment of functional dyspepsia prognosis in the Brazilian context. In this study, a relevant proportion of medical records did not allow a clear evaluation of clinical evolution, highlighting limitations inherent to the use of secondary data and reinforcing the need for more systematic clinical documentation. Furthermore, the identification of mental disorders was based exclusively on diagnoses recorded in medical records according to ICD classifications. No standardized psychiatric scales or structured diagnostic interviews were applied as part of the study protocol. Consequently, the assessment of psychiatric conditions depended on routine clinical documentation, which may have resulted in underdiagnosis, misclassification or variability in diagnostic accuracy. Additionally, the cross-sectional design prevents the establishment of causal relationships between functional dyspepsia and mental disorders, limiting temporal inferences between the conditions evaluated.

Despite these limitations, the present study is relevant, as it represents one of the few Brazilian investigations to systematically explore the association between functional dyspepsia and mental disorders in an outpatient setting. These findings contribute to a better understanding of the clinical profile of this population and support the adoption of therapeutic strategies that consider the patient in a comprehensive manner, acknowledging the close interplay between mental health and gastrointestinal symptoms.

## CONCLUSION

This study demonstrated that functional dyspepsia predominantly affects women and individuals in older age groups, reinforcing a clinical profile already described in the literature but still insufficiently explored in the Brazilian context. A high frequency of mental disorders was also observed among patients with functional dyspepsia, particularly generalized anxiety disorder and major depressive disorder, contributing to the understanding of this condition as a multifactorial disorder strongly influenced by psychosocial factors.

The coexistence of functional dyspepsia and mental disorders proved to be relevant not only from an epidemiological perspective but also from a clinical standpoint, as a substantial proportion of patients showed improvement in dyspeptic symptoms during multidisciplinary follow-up. This finding highlights the importance of integrating gastroenterology, psychiatry, and psychology in the care of these individuals, emphasizing that an approach focused exclusively on gastrointestinal symptoms may be insufficient.

Although the cross-sectional design and the use of medical record data impose limitations, the results help fill an important gap in the national literature and provide valuable insights for clinical practice in outpatient settings. By recognizing the interdependence between gastrointestinal symptoms and mental health, this study points to the need for more comprehensive therapeutic strategies that consider patients in an integral manner, respecting their biopsychosocial complexity.

Thus, strengthening multidisciplinary approaches and valuing emotional aspects in the management of functional dyspepsia may represent a significant advance in the quality of care provided, with potential positive impacts on clinical outcomes and patients’ quality of life.

## Data Availability

data not available: The data cannot be publicly shared (the database contains sensitive patient information from medical records and is subject to ethical and legal restrictions, in accordance with the Brazilian General Data Protection Law (LGPD, Law No. 13,709/2018) and the Research Ethics Committee approval)
